# Genomic sketching with multiplicities and locality-sensitive hashing using Dashing 2

**DOI:** 10.1101/gr.277655.123

**Published:** 2023-07

**Authors:** Daniel N. Baker, Ben Langmead

**Affiliations:** Department of Computer Science, Johns Hopkins University, Baltimore, Maryland 21218-2683, USA

## Abstract

A genomic sketch is a small, probabilistic representation of the set of k-mers in a sequencing data set. Sketches are building blocks for large-scale analyses that consider similarities between many pairs of sequences or sequence collections. Although existing tools can easily compare tens of thousands of genomes, data sets can reach millions of sequences and beyond. Popular tools also fail to consider k-mer multiplicities, making them less applicable in quantitative settings. Here, we describe a method called Dashing 2 that builds on the SetSketch data structure. SetSketch is related to HyperLogLog (HLL) but discards use of leading zero count in favor of a truncated logarithm of adjustable base. Unlike HLL, SetSketch can perform multiplicity-aware sketching when combined with the ProbMinHash method. Dashing 2 integrates locality-sensitive hashing to scale all-pairs comparisons to millions of sequences. It achieves superior similarity estimates for the Jaccard coefficient and average nucleotide identity compared with the original Dashing, but in much less time while using the same-sized sketch. Dashing 2 is a free, open source software.

With rapid growth of genomic databases and improvements in sequencing and assembly, there is an increasing need for efficient methods for comparing genomic sequences. Alignment-based methods are usually accurate but computationally expensive. An alternative is “genomic sketching,” a computational strategy for taking a very long sequence or collection of sequences and distilling it to a much smaller data structure. A single bit of “sketch” data structure might be standing in for many millions of bases of input data, for example. Despite being small, sketches are remarkably useful, allowing users to ask various cardinality- and similarity-related questions using only the sketch, without having to return to the larger input files.

For this reason, sketching methods based on MinHash (Broder 1997) and HyperLogLog (HLL) (Flajolet et al. 2007) have become key building blocks for scaling sequence comparison. Sketches built over all the *k*-mers in a sequence have been applied in clustering (Ondov et al. 2016), phylogenetic inference (Criscuolo 2020), strain-level profiling (Dilthey et al. 2019; LaPierre et al. 2020), species delineation (Gostinčar 2020), and summarization of genomic collections (Baker and Langmead 2019; Ondov et al. 2019).

Although existing tools like Mash (Ondov et al. 2016) and Dashing (Baker and Langmead 2019) can easily cluster tens of thousands of genomes, relevant biological data sets can reach millions of sequences and beyond. Further, these tools fail to consider multiplicities of the *k*-mers, limiting their applicability in settings in which quantities matter, for example, when analyzing collections of sequence reads or summaries from quantitative sequencing assays.

Dashing 2 builds on the recent SetSketch structure (Ertl 2021). SetSketch is related to HLL but replaces the HLL's leading zero count (LZC) operation with a truncated logarithm of adjustable base. This addresses a major disadvantage of the HLL as implemented in Dashing, because the LZC wastes ∼2 bits of space out of every 8-bit estimator (“register”) stored. SetSketch also has similarities to multiplicity-aware approaches like BagMinHash (Ertl 2018) and ProbMinHash (Ertl 2022). All three approaches (SetSketch, BagMinHash, and ProbMinHash) make decisions about whether and how to update registers by performing a random draw from a distribution, in which the draw is seeded by a hash value derived from the input item. This allows SetSketch to perform multiplicity-aware sketching in the same way as the other sketches. As an example of how users can leverage multiplicity-awareness, Dashing 2 can sketch and compare sets of genomic intervals representing RNA splice junctions, each with an associated measurement indicating how frequently the junction is observed in a particular RNA-seq experiment. Many experiments could then be compared and clustered according to the similarity of their splicing profiles.

SetSketch also admits simple and accurate algorithms for computing cardinality from a sketch, as well as computing similarity between two data sets from their sketches in a joint fashion.

Beyond advances that come with using SetSketch, Dashing 2 uses locality-sensitive hashing (LSH) to scale all-pairs comparisons to very large inputs. It finds near neighbors by grouping samples with equal register groupings. This makes Dashing 2 particularly effective for all-pairs comparisons over large sequence collections.

Dashing 2's implementation of the SetSketch structure is efficient and versatile. Like the original Dashing software, Dashing 2 can be run in a mode that sketches a sequencing data set and saves the result to a file. This is activated by the dashing2 sketch command. Also, like Dashing, Dashing 2 can compare sequences or sketches in an all-pairs fashion (dashing2 cmp or, equivalently, dashing2 dist). When combined with the ‐‐cache option, Dashing 2 loads pre-existing sketches from disk, making the command much faster. When the input to these commands consists of many sketches or data sets, Dashing 2 performs all-pairs comparisons and outputs tabular results. Dashing 2's new LSH-assisted all-pairs comparison mode can be activated via the ‐‐similarity-threshold *x* option, where, for example, *x* = 0.8 instructs Dashing 2 to use an LSH approach to consider only pairs whose similarity is likely to be ≥80%.

Dashing 2 supports a range of sequence alphabets, including the 2-bit DNA alphabet and a standard 20-letter amino acid protein alphabet (using ‐‐protein option) and compressed amino acid alphabets of size 14, eight, and six (‐‐protein14, ‐‐protein8, and ‐‐protein6) as described by Edgar (2004). Compressed protein alphabets are more appropriate when sequence identity is low.

Dashing 2's new multiplicity-aware sketching mode can be enabled for sequencing data inputs via the ‐‐prob option. Dashing 2 can sketch bigWig inputs (Kent et al. 2010) encoding numerical coverage vectors using the ‐‐bigwig option. The more generic ‐‐wsketch mode can sketch inputs consisting of keys and weights.

In addition, the modes discussed above, Dashing 2 has modes for computing containment coefficients, symmetric containment coefficient, and intersection size. Further, Dashing 2 has modes for computing Jaccard coefficients in an exact manner, without sketching or estimation; this is useful for evaluation but comes at the expense of longer running time and larger memory footprint compared with those of sketching-based approaches.

In short, Dashing 2 and the SetSketch impart a number of theoretical and engineering advances. The theoretical advances are its accuracy in its cardinality and similarity estimates, its multiplicity awareness, and its ability to scale to very large-scale all-pairs comparisons with LSH. The engineering advances of Dashing 2 enable the SetSketch approach to operate efficiently through efficient evaluation of logarithms and through use of vector instructions.

In this study, we describe the new Dashing 2 method and show its performance relative to other sketching methods. We show that Dashing 2 achieves superior similarity estimates for the Jaccard coefficient and average nucleotide identity (ANI) compared with Dashing and other tools while using the same-sized sketch. We also compare its computational performance to competing methods, showing that Dashing 2 is often the fastest tool available, especially for similarity estimation.

## Results

We used Dashing 2 v2.1.11-10-g128c. All experiments were performed on a Lenovo ThinkSystem SR630 with 48 3.0-GHz Xeon CPUs and 1.5 TB of memory.

We downloaded the RefSeq database on Jun 30, 2022 (O'Leary et al. 2016). Filtering to just complete genome sequences, we gathered 128,827 sequences, 729 from the “archea” category, 115,548 from “bacteria,” 338 from “fungi,” two from “human” (the GRCh38 and the CHM13 assemblies), 267 from “invertebrate,” 145 from “plant,” 88 from “protozoa,” 183 from “vertebrate_mammalian,” 274 from “vertebrate_other,” and 11,253 from “viral.” The compressed FASTA files occupied 475 GB. Overall, genome lengths varied from 223 bp to more than 34 billion bp, with mean and median lengths of 11.1 million bp and 4.22 million bp, respectively.

Some experiments required high-fidelity sketches. We computed a 1-MB sketch for each using Dashing 2 with sketch ‐‐binary-output -S 20 -k 31. For all 128,827 complete genomes, this process took 50 min:45 sec using GNU parallel (Tange 2011) for multiprocess parallelism.

The following subsections use these assemblies as a starting point. In particular, sections “Similarity estimation” and “ANI estimation” use sets of 1010 genome pairs selected to cover a range of similarities. The section “Large-scale sketching and pairwise comparisons” uses a subset of 50,000 assemblies to compare Dashing 2's sketching and pairwise similarity speed to that of Dashing 1. The exact lists of accessions used in each experiment are provided in files referenced in the “Software availability” section.

### Similarity estimation

We compiled a collection of pairs of assemblies covering a range of Jaccard coefficients, estimated using high-fidelity sketches. If *A* and *B* are sets of canonicalized *k*-mers from two assemblies, the Jaccard coefficient J(A,B)=|A∩B|/|A∪B|. We first performed an all-pairs comparison using the 128,827 high-fidelity sketches described above. We then partitioned the space of Jaccard estimates into 100 buckets of equal size; namely, one bucket spanned Jaccard values in the range [0, 0.01), the next spanned values in (0.01, 0.02], etc. We added an additional bucket for pairs with *J*(*A*, *B*) = 1. For each bucket, we randomly selected 10 genome pairs having a Jaccard coefficient estimate within the bucket's range. We limited our attention to RefSeq assemblies from the “archea,” “bacteria,” and “viral” groups. At the end of this process, we had a collection of 1010 genome pairs (10 for each of the 101 buckets) with Jaccard coefficients spread evenly across the range [0, 1].

To obtain a notion of “truth” to compare against, we used Dashing 2's full-accuracy mode (which does not use sketching) to compute true Jaccard coefficients for all genome pairs for both *k*-mer lengths. We also computed ANIs for all selected genome pairs using FastANI v1.33 (Jain et al. 2018). FastANI computes an approximation, so we do not call these “true” ANIs. But these have the advantage of being calculated using a separate approach from the one used to compute the Jaccard coefficients.

Using these genome pairs annotated with true Jaccard coefficients and FastANI-estimated ANIs, we compared the accuracy of Dashing 2's estimates to those of Dashing 1 v1.0 (Baker and Langmead 2019), Mash v2.3 (Ondov et al. 2016), Sourmash v4.6.1 (Brown and Irber 2016), and BinDash v1.0 (Zhao 2018). We ran Dashing 2 in two configurations. **D2** used the “one-permutation” SetSketch, with each update affecting at most one register. **D2-full** used the full update rule. We did not run the **D2W** configuration of Dashing 2 here because the goal of these experiments is to assess how well these modes estimate the typical “flat” version of the Jaccard similarity, rather than the weighted version estimated by **D2W**.

To obtain Jaccard similarities for all genome pairs, we ran bindash dist with the ‐‐mthres=1e9 option, which ensures it outputs similarities for all genome pairs not only for those with higher similarities.

Table 1 shows the sum of squared errors (SSE) between the tool-estimated Jaccard coefficient and the true Jaccard, totaled across all 1010 genome pairs. We show these results for sketch sizes of 8 kbits (8 × 1024 bits, equivalent to 1 kilobyte), 32 kbits, and 128 kbits. Comparisons across tools and modes use sketches of the same total size in bytes. We note that although we set the total sketch size constant across tools, the tools use different register sizes according to their sketching algorithm. Mash and Sourmash use 64-bit registers for example, whereas Dashing and Dashing 2 used 1-byte registers. In all cases, either D2 or D2-full achieved lowest SSE, with the other achieving second-lowest. Dashing 2 and Dashing 1 both achieved lower SSE than Mash and Sourmash. Unlike Dashing 1, Dashing 2's modes consistently yielded better Jaccard coefficient estimates than BinDash.

**Table 1.**
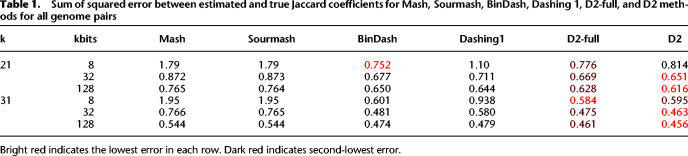
Sum of squared error between estimated and true Jaccard coefficients for Mash, Sourmash, BinDash, Dashing 1, D2-full, and D2 methods for all genome pairs

We note that the increased accuracy of Dashing 2 over Dashing 1 is expected, because the flexibility in its log base allows it to make the best possible use of the bits allocated to each register. For details on how Dashing 2 makes more efficient use of register space, see Methods (section “SetSketch”).

In these experiments, we used Sourmash in a way that achieved the same overall sketch size as the other methods. Because Sourmash's default is to build a sketch proportional in size to the input, this required that we configure it to use a fixed-size sketch via its sketch dna -p num=X option, with X equal to the number of 64-bit registers needed to match the other sketches.

### ANI estimation

We further assessed the ANI estimates obtained by using the Mash distance equation and rescaling: *ANI*_est_ = 1 + 1/*k* · ln(2*J*/(1 + *J*)). Here *k* is the *k*-mer length, and *J* is the estimated Jaccard coefficient. Negative *ANI*_est_ values were rounded up to zero. In this experiment, we additionally assessed the new multiplicity-aware (“weighted”) mode of Dashing 2, called **D2W**. The feature-hashing structure needed to obtain the weights for **D2W** mode was configured to consist of 5 million 64-bit counts.

In this case, the input *J* to the *ANI*_*est*_ equation was the probability Jaccard similarity (*J*_*P*_) described in section “Weighted SetSketch,” rather than the typical “flat” Jaccard coefficient. This experiment allows us to assess whether *J* (as estimated by Dashing 1, D2, or D2-full) or *J*_*P*_ (as estimated by D2W) yields a better ANI estimate.

We assessed SSE between *ANI*_*est*_ and the FastANI-estimated ANIs for Mash, Dashing 1, D2, and D2W (Table 2). In all cases, the D2W approach achieved either the lowest or second-lowest SSE, with either D2 or Dashing 1 achieving the second-lowest SSE.

**Table 2.**
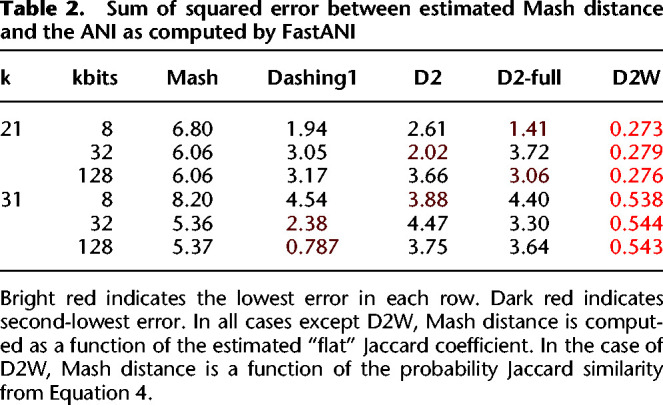
Sum of squared error between estimated Mash distance and the ANI as computed by FastANI

We did not compare to Sourmash in this experiment because Sourmash's preferred method for estimating ANI uses a scaled sketch, namely, a sketch whose size grows as a function of the length of the input sequence. Because Dashing 2 operates using a given, fixed sketch size, we designed a separate experiment to compare Dashing 2 with Sourmash on ANI estimation, presented in the following section.

### Comparison to Sourmash ANI estimation

Sourmash (Brown and Irber 2016) has a specific facility estimating the ANI between two sketches when the sketches are built in a “scaled” fashion, in which the number of hash values retained is proportional to the length of the input sequence. We conducted a separate experiment to compare the ANI estimates from Sourmash's “scaled” mode to Dashing 2's D2, D2-full, and D2W's modes.

Unlike Dashing 2, Sourmash's scaled sketch does not have a fixed size; rather, the number of registers is a function of the input sequence length. To make the comparison fairer, we first ran the Sourmash sketch dna command in its default mode, which builds a scaled sketch, on each of the 1010 pairs of FASTA files used in the previous experiments. We then determined the number of 64-bit registers (“signatures” using Sourmash's term) included in each sketch, which can be performed by loading and interrogating the sketch file in Python. We then built Dashing 2 D2, D2-full, and D2W sketches for each FASTA, configuring Dashing 2 to use the same-sized sketch (in total bits) as Sourmash did for that FASTA. However, because Dashing 2 can only estimate Jaccard (or ANI) between equal-size sketches, we sketched both FASTAs in each pair using the size of the *smaller* of the two Sourmash sketches for the pair. In this way, the Sourmash sketches were always at least as large as the Dashing 2 sketches, and sometimes larger. Although this puts Dashing 2 at a mild disadvantage, it still produces more accurate ANI estimates overall, as seen in Table 3.

**Table 3.**
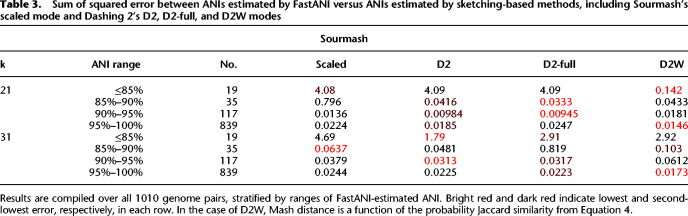
Sum of squared error between ANIs estimated by FastANI versus ANIs estimated by sketching-based methods, including Sourmash's scaled mode and Dashing 2's D2, D2-full, and D2W modes

### Large-scale sketching and pairwise comparisons

We used Dashing 1 and Dashing 2 to sketch a sample of 50,000 complete genome assemblies downloaded from RefSeq. Both were run using GNU parallel, allowing up to 12 sketching processes to run at a time. Both tools were configured to produce sketches 1 MB in size. Dashing 1 took 2 h:00 min:34 sec to construct all the sketches, and Dashing 2 took 50 min:46 sec.

We then used Dashing 1 and 2 to perform exhaustive all-pairwise Jaccard similarity comparisons across the 50,000 genomes (two human assemblies and 49,998 bacterial assemblies), comparing a total of 1.25 billion pairs of 1-MB sketches. Both tools used their default similarity estimation methods. In the case of Dashing 1's, this was the MLE estimator of Ertl (2017). In the case of Dashing 2, this was the simple joint estimator described in the section “Similarity comparison.” Both Dashing 1 and Dashing 2 were run with the -p80 ‐‐presketched options, enabling 80 simultaneous threads of execution and instructing both tools to use the already-computed sketches. Dashing 1 took 49 h:41 min to estimate all-pairwise similarities, whereas Dashing 2 took 5 h:41 min and was about 8.7 times faster.

### Speed and memory footprint comparison

We compared several sketching tools with respect to their running time and memory footprint when sketching and performing all-pairs comparisons across the same 2020 genome assemblies (1010 pairs) used in the similarity estimation results above. Results are given in Supplemental Table S1. We found that Dashing 2's modes perform well in all categories. It particularly excels at all-pairs similarity comparison running time. BinDash has the lowest memory footprint for sketching, and Mash and BinDash are both competitive with Dashing 2's modes for sketching running time.

### All-pairs comparisons using LSH

We performed all-pairs comparisons for a large collection of proteins by combining SetSketch, using the LSH scheme described in section “LSH implementation” to avoid comparisons unlikely to meet a minimum similarity threshold. We used the UniProtKB/Swiss-Prot collection of 565,254 protein sequences, v2021_03. For each protein, we used Dashing 2 to create a 10-mer sketch (-k10) of 256 registers (-S256). Proteins were translated to a 14-letter reduced alphabet (‐‐protein14) to capture more distant homology (Edgar 2004). We ran Dashing 2 in sketch ‐‐topk mode to perform all-pairs comparisons while avoiding pairings that fail to appear in the top 256 neighbors of a protein. We used the default of ‐‐nLSH 2 to use two distinct sizes of superregister groupings, corresponding to the *N* parameter of Algorithm 2. The output was a *k*-nearest-neighbor (KNN) graph in tabular (TSV) format, associating each protein to the 256 others with greatest Jaccard coefficient. We used 80 simultaneous threads for these experiments.

Because of both estimation and LSH error, the graph may include false positives (reported neighbors that are not truly among the top 256) and/or false negatives (unreported neighbors that are truly in the top 256). To measure the error introduced by LSH, we compared the KNN graph to another KNN generated using exhaustive all-pairs comparisons. Importantly, the exhaustive KNN was also built using Jaccard coefficient estimates, because exact computation of the Jaccard coefficient is too computationally expensive. Thus, this experiment isolates error owing to the LSH filter only and does not assess error owing to the Jaccard estimate.

Relative to the exact method, Dashing 2's LSH method achieved 100% recall and precision; namely, there were no false positives or false negatives among the neighbors found using LSH filtering. Further, using the LSH, Dashing 2 was able to generate the graph in 43 sec compared with 56.2 min for the exhaustive method, a 78-fold speedup. The exhaustive method ultimately performed 159,755,759,631 pairwise comparisons, compared with approximately 470 million comparisons performed by the LSH-assisted method. The 470 million comparisons performed by LSH is about 3.5 times greater than the minimum determined by the number of neighbors (256) times the number of proteins (565,254).

We also applied this approach to create a nearest-neighbor graph over the larger UniRef50 data set, which is built by clustering UniRef90 seed sequences from the UniProt Knowledgebase v2021_03 having at least 50% sequence identity to and 80% overlap with the longest sequence in the cluster. The database contains 53,625,855 sequences totaling over 15 billion amino acids. After sketching, the KNN graph was generated in <10 min. In contrast, the exhaustive all-pairs comparisons approach required a much longer amount of time; we interrupted this computation after 2 d and extrapolated that the total time would have exceeded a year.

We note that although there are tools for large-scale clustering that use similar ideas, like Linclust (Steinegger and Söding 2018), no other genomic sketching method (Mash, BinDash, Sourmash) currently has this ability.

## Discussion

Dashing 2 combines the SetSketch with LSH to bring multiplicity-aware sketching and fast filtering to genomic sketching analysis. Its all-pairs comparison method scales effectively to millions of sequences. Dashing 2 can sketch FASTA and FASTQ inputs, as well as protein-sequence inputs using a variety of protein alphabet reductions. It can compare sequences based on Jaccard coefficient, Mash distance, or containment coefficient. It can also compare sequences based on a weighted version of the Jaccard coefficient that is aware of the multiplicities of the input items.

Dashing 2's estimation error for the Jaccard coefficient and ANI estimates was lower than that of the previous version of Dashing and was substantially lower than that of Mash. Dashing 2 had lower or comparable error to BinDash. Thus, it will be important to continue to study the SetSketch as a highly accurate alternative not only to the HLL sketch but also to the MinHash and b-bit MinHash methods.

Notably, Dashing 2's ANI estimation error was lowest when taking multiplicities of the input items into account, namely, when using a weighted Jaccard coefficient as input to the Mash distance equation. This shows that multiplicities are helpful not only for quantitative applications but also in typical “flat” sketching scenarios. This motivates future study of sketch data structures that account for multiplicities, as well as methods—like Dashing 2's feature hashing method—for efficiently compiling multiplicity information before sketching.

We have concentrated on comparing SetSketch to the more commonly used methods of MinHash (as implemented in Mash and Sourmash), HLL (as implemented in Dashing 1), and b-bit MinHash (as implemented in BinDash). But there are other sketching approaches that have been proposed with which it may also be relevant to compare, including HyperMinHash (Yu and Weber 2022).

The term “sketch” is used for various data structures in Bioinformatics. Dashing 2 is particularly designed for scenarios in which many sequencing data sets must be compared with each other in an efficient, scalable way. For this scenario, we use the expedient of modeling sequence data as a set (or multiset) of *k*-mers, which is reduced to a sketch of fixed size, for example, 1 kilobyte. Other studies describe approaches that use some form of sketching, but that keep more detail about the sequence content of each data set, for example, by keeping an ordered sequence of representative subsequences from the longer sequence (Marçais et al. 2019; Jain et al. 2020). Other studies focus on only pairwise or one-versus many queries (Jain et al. 2018; Ondov et al. 2019; Shaw and Yu 2023). Such tools can be a better fit in situations in which there is good reason to treat the sequences in very small batches (e.g., pairwise comparisons) or to treat them asymmetrically (e.g., one-vs.-all queries). But Dashing 2 particularly excels when many are compared in a symmetric, all-pairs fashion.

We note that Dashing 2's LSH mode is intended to facilitate scalable all-versus-all comparisons among sequences in a large collection. This is distinct from the purpose of tools like mash screen, which seek to estimate similarity (more specifically, containment) between a single query and many reference sequences in a database. In short, whereas mash screen applies sketching to accelerate one-to-many comparison problems, the new methods described here are designed to accelerate many-to-many comparisons.

Finally, we note that although genomic sketching tools have typically been applied to compare sequences or sets of sequences, they are also useful for comparing other inputs, such as genomic intervals or other very large sets of string identifiers. Dashing 2 takes a step toward enabling this large set of applications by allowing for BED and LeafCutter inputs.

## Methods

### Background

A sketch distills a large set of items into a small data structure while preserving the ability to estimate quantities like the data set's cardinality, namely, how many distinct items/*k*-mers are present. Sketches for different data sets can also be compared to estimate their similarity. This can be performed with far less memory or in far less time compared with that required to compare the original, unsketched data sets.

Genomic data usually take the form of long sequences, so we must first convert the sequence to a set. Usually, we decompose the sequence into the set of its constituent length-*k* substrings, namely, its *k*-mers. Because sequences that are reverse complements of each other should be considered identical, *k*-mers are usually canonicalized: If a given *k*-mer is greater than its reverse complement, it is replaced with its reverse complement.

The three sketch types most relevant to this work are MinHash (Broder 1997), HLL (Flajolet et al. 2007), and the recent SetSketch (Ertl 2021). All three consist of an array of registers, in which a register is an instance of a “high-water-mark” random variable (RV for short). The nature and form of a high-water-mark RV differs between sketch types, but its purpose is to record the most “extreme” input item observed so far. Second, all three sketch types have an update rule that executes once per input item and that can update the values of zero or more registers. Third, all the sketch types have associated algorithms for estimating summary quantities of interest, including cardinality and Jaccard similarity, from the register values. These algorithms differ across sketch types, both in their details and in their statistical guarantees. Finally, all of these sketch types are composable, in the sense that two sketches of the same type (and same hashing scheme and size) created for data sets *A* and *B* can be combined to obtain a sketch for the data set A∪B through a simple combination of their register values.

The sketch types differ in the nature of their high-water-mark RVs and, consequently, their update rules and summary algorithms. Practical MinHash implementations like Mash use a bottom-*k* approach; a hash function is applied to each input item, and then the *m* sketch registers are set to the *m* smallest distinct hash values obtained. In this way, registers act as order statistics (minimum, second minimum, third minimum, K, *m*th minimum) over uniform random draws seeded by the input items. The update rule simply involves hashing the new input item and, if its hash value is less than the *m*th minimum currently in the sketch, updating the sketch to include the new value. Algorithms for estimating cardinality and Jaccard similarity using bottom-k MinHash are simple, and their statistical guarantees are shown elsewhere (Cohen 2014; Hassanian-esfahani and Kargar 2018).

HLL implementations use a *k*-partition approach, rather than the bottom-*k* approach. Each item is hashed, and its hash value is partitioned into a prefix *p* and a suffix *q*. The prefix *p* determines which register (“partition”) the item maps to, whereas the suffix *q* determines how the register should be updated. Specifically, the algorithm finds the number of consecutive unset bits in the most significant digits of *q*, namely, its LZC. If the LZC is greater than the register's current value, the value is set to the LZC. Because the individual bits of the hash values can be considered independent Bernoulli(0.5) draws, the HLL high-water-mark RVs are essentially taking the maximum over many Geometric(0.5) draws. Algorithms for estimating cardinality and Jaccard similarity using HLL are relatively more complex, sometimes with fewer statistical guarantees compared with the MinHash estimators (Ertl 2017).

### SetSketch

Dashing (Baker and Langmead 2019) used the HLL data structure and its LZC strategy to update register values, as described above. Whereas Dashing's HLL registers were each 8 bits wide and able to hold a value in the range zero to 255, LZCs could range only from zero to 64. In fact, LZCs would usually span a smaller range than this, because bits used for the prefix *p* are not considered. An LZC would therefore fail to use at least 2 bits of an 8-bit register, leaving the structure 25% empty. Although registers could be shrunk to 6 bits, this would conflict with Dashing's use of SIMD instructions with 8-bit operands.

Ertl's SetSketch (Ertl 2021) addresses this issue by replacing the LZC with a logarithm of configurable base *b*. This comes with a drawback: The addition of a single item to the SetSketch potentially updates the values of *all* registers, rather than just one. Given a data item *d*, the update rule for each register *K*_*i*_, is

(1) $$K_{i}=\max(K_{i},\lfloor 1-\log_{b}h_{i}(d))\rfloor ),$$

where *h*_*i*_ is an independent hash function specific to register *i* distributed exponentially; namely, $h_{i}(d)∼\mathrm{Exp}(a)$. An illustration of this sketch building is shown in Supplemental Figure S1.

For reasons explained below, it is useful to factor this update rule into two phases, with delaying the logarithms to the second phase. We use *K*_*i*_ to denote the register's value at update time and $K_{i}^{\prime }$ to denote its value after the final truncation:

(2) $$K_{i}=\min(K_{i},h_{i}(d))\;K_{i}^{\prime }=\lfloor 1-\log_{b}K_{i}\rfloor .$$

where again $h_{i}(d)∼\mathrm{Exp}(a)$. The subtraction in the $K_{i}^{\prime }$ formula inverts the notion of “extremeness” from a minimum to a maximum, hence the use of min in Equation 2 versus the use of max in Equation 1.

The strategy of letting each register's value be a function of all the input items has advantages. First, because the final value is a function of all items rather than a register-specific subset of them, the final values are statistically independent. Second, the exponential rate *a* and logarithm base *b* are parameters of the sketch. We can set *b* in a way that spreads the *K*_*i*_ values over a range of our choosing. If registers are 8 bits wide, we can choose *a* and *b* so that $\left\lfloor 1-\log_{b}h_{i}(d))\right\rfloor$ ranges from zero to 255, landing outside the range only with low (and controllable) probability. If registers are another size (e.g., Dashing 2 supports 4-, 8-, 16-, 32-, and 64-bit registers), *a* and *b* can be adjusted. Methods for setting *a* and *b* are described in Section 2.2 of Ertl (2021).

This comes with a potential disadvantage. Because each register is a function of every input item, each addition may require *O*(*m*) work, where *m* is the number of registers. Ertl (2021) proposes optimizations that ensure that the work per update quickly becomes *O*(1) in the typical case in which the input is much larger than *m*. This is accomplished by (1) maintaining a value *K*_max_ equal to the maximum among all the registers’ current values, and (2) reordering the inner loop so that iterations occur in increasing order by value of the $h_{i}(d)∼\mathrm{Exp}(a)$ draw. Note that the maximum is maintained over the original, untruncated exponential draws in the *K*_*i*_ variables, not the truncated version eventually stored in the $K_{i}^{\prime }$ variables. Once we reach an iteration in which the draw has value *h*_*i*_(*d*) > *K*_max_, neither the current nor a subsequent iteration can possibly change the value of a register, and the inner loop can break.

Another disadvantage of the SetSketch is the computational cost of the exponential draws and, related to that, the computational cost of logarithms. Although the strategy just described reduces the number of exponential draws, later sections describe how we reduce the cost of logarithms.

### Similarity comparison

Dashing 1's default algorithm for estimating the Jaccard coefficient between HLLs *A* and *B*, *J*(*A*, *B*) used the maximum likelihood estimation (MLE) method of Ertl (2017). This models register values as Poisson random variables and uses an iterative root-finding to estimate the Poisson parameter from the histogram of register values in the union (A∪B) HLL. This was less accurate but substantially faster than the related joint MLE (JMLE) method (Ertl 2017), which required histogramming of joint register values.

Dashing 2 uses a simpler joint estimator named ${\hat{\mu }}_{\mathrm{simple}}$ (Ertl 2021). (Note that this estimator is described only in the “v1” version of the Ertl [2021] paper preprint-cited. Later versions of the paper describe Brent's root-finding algorithm instead.) It has a closed-form solution and does not require an iterative root-finding procedure. Being a “joint” estimator, ${\hat{\mu }}_{\mathrm{simple}}$ also does not require a union sketch; it is a function only of the input sketches *A* and *B*. Finally, ${\hat{\mu }}_{\mathrm{simple}}$ does not require a histogram of register *values*. Instead, it requires two counts: *D*_+_ and *D*_−_. *D*_+_ is the number of registers in *A* that are greater than their counterparts in *B*, and *D*_−_ is the number of registers in *A* that are less than their counterparts. Unlike MLE and JMLE histograms, *D*_+_ and *D*_−_ can be computed using only single-instruction multiple-data (SIMD) instructions. In particular, a combination of SIMD greater-than/less-than and population-count instructions enable rapid tallying of *D*_+_ and *D*_−_ with respect to chunks of registers at a time. As described by Ertl (2021), a mathematical complication arises when the sets are mostly disjoint. We fall back on Ertl's alternative formulae α_disj_ and β_disj_ from Ertl (2021) in such cases.

### Full Dashing 2 sketch update

Algorithm 1 gives the update algorithm for the full version of the Dashing 2 SetSketch. This is the default for weighted sketching and can be enabled for nonweighted sketching using the option ‐‐full-setsketch. Without this option, the one-permutation strategy described in the next subsection is used instead. Inputs consist of the register array *K* comprising the sketch, a MaxTree array *T* maintaining maxima over power-of-two-sized stretches of registers, the item *X* to be added, and the item's weight *W*. When used for unweighted sketching, *W* equals one. Registers in *K* are initialized to the maximum possible value.

Algorithm 1. **Update full Dashing 2 SetSketch.**

** Input:** SetSketch *K*[0..*m* − 1], MaxTree *T*[0..*m* − 2], item *X* of weight *W*

** Result:** *K* and *T* updated according to *X*, *W*

** 1** RNG ←RandomNumberGenerator(*X*, *m*)

** 2** RV ←RNG.nextExponentialSpacing(0, *m*, *W*)

** 3** *K*_max_ ← T.max()

** 4** **if** *RV*>*K*_*max*_ **then**

** 5**  **return**

**  end**

** 6** *i* ← RNG.nextFisherYates()

** 7** **if** *RV*<*K*[*i*] **then**

 **8**  *K*[*i*] ← RV

 **9**  *K*_max_ ← T.update(*K*[*i*], *i*)

  **end**

**10** **for** *i* ← 1, *m* − 1 **do**

**11**  RV ←RV + RNG.nextExponentialSpacing(*i*, *m*, *W*)

**12**  **if** RV > *K*_*max*_ **then**

**13**   **return**

  **end**

**14** i ←RNG.nextFisherYates()

**15** **if** *RV*<*K*[*i*] **then**

**16**  *K*[*i*] ← RV

**17**  *K*_max_ ← T.update(*K*[*i*], *i*)

  **end**

 **end**

A single update could require modifying any number of registers, from zero to *m*. To avoid *O*(*m*) work on average, the algorithm follows the strategy of Ertl (2021), examining registers in order according to the probability they will be updated, namely, according to the extremeness of that register's exponential random draw. The algorithm uses a pseudorandom number generator RNG, seeded with *X* for deterministic updates. RNG.nextExponentialSpacing (*i*, *m*, *W*) returns the value that must be added to obtain the next draw in increasing order, according to the recurrence $x_{i}=x_{i-1}+\frac{1}{m-i+1}\mathrm{Exp}(aW)$. The recurrence follows from the memoryless property of the exponential distribution (Ertl 2022). RNG.nextFisherYates() samples a new register randomly without replacement with Fisher–Yates shuffling, as in algorithm 6 by Ertl (2022). By matching the increasing series of exponential draws with a random sequence of register choices, we visit registers in the desired order of most to least likely to be updated. Kahan summation (Kahan 1965; Babuska 1969) is used to reduce numerical errors when summing exponential spacings.

Conditional statements in lines 4 and 12 of Algorithm 1 abort upon reaching a draw that is less extreme than the least extreme that could affect a register value (*K*_max_). Accordingly, the *K*_max_ ← T.update(*K*[*i*], *i*) statements in lines 9 and 17 update a structure maintaining the current “least extreme draw” in the *K*_max_ variable per algorithm 4 of Ertl (2022). Updates to the *K*_max_ structure take *O*(log (*m*)) time and are required only when a register is modified. The number of loop iterations is inversely related to the number of items added, approaching zero as the number far exceeds *m*.

### One-permutation SetSketch

By default, Dashing 2 computes unweighted sketches and uses an economical “one-permutation” update method (Wang et al. 2017), which modifies at most one register per update. This method uses some bits of the random draw to choose which register to update (Supplemental Material, Algorithms S2 and S4), similar to how the HLL update rule in Dashing uses the hash prefix *p*.

Although the one-permutation approach is efficient compared with a full update, accuracy suffers when many registers are empty, namely, when the input has few items relative to *m*. To maintain accuracy, we implement the densification approach of Shrivastava (2017), applied after finalization. This strategy has similar accuracy compared with the full SetSketch but is more efficient in practice. Because of this, we made this one-permutation mode the default for unweighted sketching. The full update rule is used instead when the user enables weighted sketching or when the user enables it with the ‐‐full-setsketch option.

### SetSketch parameters

Like the HLL, the number of registers *m* is a parameter of the SetSketch. Unlike HLL, SetSketch has a related parameter, the register *width* in bits. Other key parameters are the rate for the exponential draws (*a*), and the log base used for truncation needed to fit draws into registers (*b*). As in the work by Ertl (2021), we use *q* to denote the value that is one less than the maximum register value; for example, for 8-bit registers, *q* = 2^8^ − 1 − 1 = 254.

Although Ertl (2021) gives theoretical guidelines for choosing *a*, *b*, and other parameters, these assume foreknowledge of input cardinalities. On the other hand, multiple SetSketches are comparable only if they were built using identical parameters. This creates a tension between wishing to choose the parameters sooner, in order to make compact sketches, versus later, to delay truncation until we can ensure all relevant sketches are constructed and truncated with identical *a*, *b*, and *q*.

Dashing 2's default strategy is to set *a* and *b* according to the overall set of input data sets and to shape and truncate the sketches according to user-configurable choices for *m* and *q*. Dashing 2 also allows the user to delay the choices for *a*, *b*, and *q*, so that larger, untruncated sketches can be stored temporarily in preparation for future truncation and comparison with other sketches.

To select *a* and *b* according to the data, Dashing 2 first forms untruncated sketches and then computes *b* and *a* according to the expression

(3) $$b=\exp\left(\frac{\ln\;\max(K^{*})/\min(K^{*})}{q}\right)\;a=\max(K^{*})/b,$$

where K* denotes a concatenations of all untruncated register values from all inputs. For experiments in this study, we invoked Dashing 2 with all input data sets at once, ensuring *a* and *b* are set identically for all.

Two other parameters to discuss are the *k*-mer length used when the input is a sequence and the total number of registers in the sketch, *m*. Dashing 2's default *k*-mer length is the largest such that the representation of a *k*-mer fits in a 64-bit unsigned variable. For DNA this is 31, and for protein, this is 14 (or greater for reduced protein alphabets). The default sketch size *m* is 1024.

### Delayed logarithms

Potentially expensive logarithm calculations are used in two tasks: (1) truncation of register values and (2) to perform the exponential draws. Dashing 2 avoids these costs in two ways, described here and in the following section.

First, it uses the two-step strategy of Equation 2 to delay truncation until a finalization step, which runs only after all items are added (Supplemental Material, Algorithm S3). Before finalization, intermediate register values are stored as untruncated 64-bit floating-point numbers. As a result, the update rule uses only a minimum, rather than both a logarithm and a maximum. The total number of logarithmic truncations performed at most *m* regardless of the number of items added to the sketch.

This comes at the cost of requiring additional space at sketching time. For the entire algorithm up to finalization, we must store a 64-bit value for each register even if finalization will later reduce that to, for example, 8 bits. Because practical sketches require only thousands of registers, this is not onerous in practice.

### Approximate logarithms

The second use of logarithms is in the exponential Exp(*a*) random draw, which is accomplished by computing −ln(Unif())/*a*, where Unif() is a uniform random draw between zero and one. We observed that, once the sketch becomes quite full, many exponential draws are well above the *K*_max_ ceiling, aborting the inner loop. Although an inaccurate logarithm might cause us to miscompute whether a draw is under the *K*_max_ ceiling, this arises only for draws near the ceiling. We use a fast, approximate logarithm first, reverting to a more accurate (and expensive) logarithm only if the first result is close to *K*_max_.

For fast logarithms, we use an approximation computed using the floating-point number's integral representation. Specifically, we use a modified version of the algorithm of Schraudolph (1999). This can overestimate the result by a multiplicative factor of up to 1.42. By dividing the fast-logarithm result by this number, we can determine if the approximation is close enough to *K*_max_ to require a full logarithm computation. This affects the computation within RNG.nextExponentialSpacing(), called in lines 2 and 11, as well as the conditional checks in lines 4 and 12, of Algorithm 1, although we omitted these details from the algorithm listing.

### Weighted SetSketch

Although Ertl (2021) describes applications only to unweighted sketching, we extended SetSketch to consider weightedness using the ProbMinHash (Ertl 2022) strategy, namely, by multiplying the exponential draw's rate parameter by the item's weight, represented by argument *W* to RNG.nextExponentialSpacing() in lines 2 and 11 of Algorithm 1. When comparing sketches weighted in this way, the quantity being estimated is a version of the Jaccard coefficient called the “probability Jaccard similarity,” *J*_*P*_:

(4) $$J_{P}(A,B)=\underset{d\in D\;\;s.t.\;w_{A}(d)=w_{B}(d)=0}{\sum }\frac{1}{\sum_{d^{\prime }\in D}\max\left(\frac{w_{A}(d^{\prime })}{w_{A}(d)},\frac{w_{B}(d^{\prime })}{w_{B}(d)}\right)},$$

where *D* is the item universe, and *w*_*A*_ and *w*_*B*_ are weight functions for items in sets *A* and *B*.

A key question is how to obtain *w*_*A*_ and *w*_*B*_. When the input is sequencing data, the weight of an item (*k*-mer) should equal its relative frequency. By default, Dashing 2 will use a hash table to track the exact relative frequency for each item. But Dashing 2 also supports a faster and more memory-efficient method that estimates each item's frequency using a feature hashing (Moody 1988) approach, equivalent to a single-row Count-Min Sketch (Cormode and Muthukrishnan 2005). This mode is enabled with the ‐‐countsketch-size option. Nonsequencing data sets might also come with an inherent notion of “weight”; for instance, if input items represent genes and associated expression levels, these levels could be immediately used as weights, without the need for counting or for the feature-hashing data structure.

Optimizations for the logarithms described above are also used for weighted sketching.

### LSH implementation

To scale all-pairs comparisons, we implemented a filtering approach based on LSH. First, we note that although sketching approaches like MinHash can themselves be viewed as a form of LSH, here we use the term to refer to an approach that Dashing 2 performs after sketches are already computed. This approach's goal is to minimize the number of below-threshold pairwise comparisons performed while skipping as few above-threshold comparisons as possible.

The LSH method works by grouping SetSketch registers into “superregisters.” For instance, the first four registers (*K*[0…3]) might constitute the first superregister, the next four (*K*[4…7]) the second superregister, etc. Associated with each superregister is a map from possible values to a list of all input data sets having that value in that superregister. In our example, the keys for the first superregister will consist of all combinations of the first four registers *K*[0…3] observed in an input data set, and the values will be the associated lists of data sets. An LSH index might consist of several such tables, each with a distinct superregister group size. In the Supplemental Material, Algorithm S1 shows how the index is updated with one additional data set.

Because registers are independent, a size-*P* superregister will match between data sets *A* and *B* with probability *J*(*A*, *B*)^*P*^, where *J* is the Jaccard coefficient. When performing a large-scale all-pairs comparison, Dashing 2 begins by computing LSH indexes for values of *P* in a user-configurable subset of the values {1, 2, 4, 6, 8, 10}. By default, Dashing 2 tries *P* ∈ {1, 2}, but it can be configured to try more values for *P* via the ‐‐nlsh option, corresponding to the *N* variable in Algorithm S1 (in the Supplemental Material) and Algorithm 2. For *P* ∈ {1, 2}, the superregisters are formed by partitioning registers into *m*/*P* nonoverlapping groups. For larger values of *P*, we select a random set of *m* · 8 /*P* contiguous groups of registers. In this case, superregisters can overlap.

In the Supplemental Material, Algorithm S1 details how a single data set is added to an LSH index. Algorithm 2 details how we query to find a list of candidate nearest neighbors for a data set using the LSH index. In both cases, a pair of nested loops is used. The outer loop iterates over LSH tables from the most to least specific (largest to smallest *P*), whereas the inner loop iterates over superregisters. In the case of Supplemental Algorithm S1, an iteration of the inner loop updates the LSH table with the *id* of the current data set. In the case of Algorithm 2, an iteration of the inner loop contains a final loop that updates a running list of candidate data sets with all other data sets having the same value for the current superregister.

Algorithm 2. **Find candidates for *k*-nearest neighbors for data sets using the LSH index. The result is a list of at most three *k* candidates, which is later refined and ordered using Jaccard similarity.**

** Input :** *Ks*: Map from *id*’s to SetSketches, for all data sets

** Input :** *k*: Target number of nearest neighbors to find

** Input :** *id*: Identifier for this data set

** Input :** *RNG*: Pseudorandom number generator

** Input :** *seed*: Pseudorandom seed

** Input :** *N*: Number of superregister sizes in index

** Input :** *LSH*: map from table id, superregister id, superregister value triples to a corresponding list of data sets

** Output:** *L*: List of nearest neighbors

** 1** i ←*N* − 1

** 2** **if** *i* > 2 **then**

** 3** *RNG*.initialize(*seed*)

**  end**

 // Over tables 0.. *N*-1, from largest superregister size (most specific) to smallest (least specific)

** 4** **while** *i*≥0 **do**

** 5** *P* ←min (2^*i*^, 2*i*)

** 6** **if** *i* ≤ 2 **then**

** 7**  S ←m/ N

**   else**

** 8**  S ←m⋅8/ N

   **end**

** 9** *j* ← 0

   // Loop over superregisters

**10** **while** *j* < *S* **do**

**11**  **if** *i* ≤ 2 **then**

    // Next nonoverlapping superregister

**12**   SuperReg ←Ks[id][K⋅j..K⋅j+P−1]

   **else**

     // Random superregister of length P

**13**    *ri* ← RNG.randomInt(0,*m* − *P*)

**14**    SuperReg ←Ks[id][ri..ri+P−1]

   **end**

   // Append this data set to the list for this table, superregister

   // superregister value combination

**15**  **for** *n* ∈ *LSH[⟨i,j,SuperReg⟩]* **do**

**16**   *L*.append(n)

**17**   **if** *L*.length() = *k* · 3 **then**

**18**    **return** *L*

    **end**

   **end**

**19**   *j* ← *j* + 1

 **end**

**20** *i* ← *i* − 1

**21** **return** *L*

**end**

The LSH tables are used in two distinct modes of Dashing 2. The mode activated with (‐‐topk) builds a *k*-nearest neighbor (KNN) graph from the input data sets and follows the logic of Algorithm 2. For a given pivot genome, we use the LSH tables to generate a list of $\left\lceil O_{s}\times k\right\rceil$ candidates for each input genome, where *O*_*s*_ > 1 is an oversampling rate, set to three by default. We then estimate the Jaccard similarity between the pivot and each of the candidates in the order they were discovered, keeping only the *k* with the greatest Jaccard coefficients. Although this can result in some misreported neighbors, for example, because a near neighbor happened not to coincide with the pivot in any superregister, this possibility is reduced both by oversampling and by the order in which we attempt the LSH tables, namely, from most to least specific.

In another mode, Dashing 2 reports pairwise distances between all pairs of genomes having similarity above some threshold (‐‐similarity-threshold X). In this mode, there is no additional limit on the number of “neighbors” that might be reported for a genome. Although querying the index, we maintain a heap of all neighbors with similarity above a given threshold.

### Exact similarity mode

The ability to compute exact Jaccard coefficients is useful for evaluating Dashing 2's estimates. Dashing 2 therefore implements two modes for exact computation of Jaccard coefficients. One uses sorted *k*-mer hash sets (‐‐set); the other uses *k*-mer count dictionaries (‐‐countdict).

### Sketching sequencing reads

Dashing 2 can also sketch inputs consisting of sequencing reads, for example, in FASTQ format. This involves the extra challenge of handling sequencing errors, because *k*-mers containing errors can be far more numerous than correct *k*-mers and so can dominate and bias similarity estimates. For this reason, methods for sketching sequencing-read inputs attempt to filter out *k*-mers containing sequencing errors before computing cardinality or similarity. Dashing 2 adapts the approach of Mash (Ondov et al. 2016) for eliminating *k*-mers below a specific count threshold. For instance, if the target threshold is set to two, Dashing 2's SetSketch implementations (both one-permutation and full) maintain a dictionary of items seen fewer than two times so far. Once an item's count reaches two, it is added to the final SetSketch structure.

Dashing 2 can also use a down-sampling approach (‐‐downsample <fraction>) to randomly keep a specified fraction of the input *k*-mers. The decision to keep or suppress a *k*-mer is made independently for each *k*-mer rather than for each *distinct k*-mer. In this way, frequent *k*-mers—namely, those occurring more than $\frac{1}{F}$ of the time—are unlikely to have all copies suppressed. The filter incurs little computational cost but greatly reduces the number of error *k*-mers ending up in the sketch. This is similar to the ideas used in sequencing error correction (Song et al. 2014) to distinguish *k*-mers with or without sequencing errors.

Weighted sketching modes can be particularly appropriate for sequencing reads because they have the effect of down-weighting error *k*-mers, which tend to occur infrequently compared with correct *k*-mers.

### RefSeq data sets

A list of the accessions for the 1010 RefSeq genome pairs used in the section “Similarity estimation” is available at https://www.cs.jhu.edu/~langmea/resources/d2/pairs1010.csv.

Accessions for the 50,000 RefSeq genomes used in the experiments in the section “Large-scale sketching and pairwise comparisons” are available at https://www.cs.jhu.edu/~langmea/resources/d2/refseq50k.txt.

### Software availability

Open source code for the Dashing 2 software is available as Supplemental Code and at GitHub (https://github.com/dnbaker/dashing2). Scripts for performing the experiments described in this paper are available as Supplemental Material and at GitHub (https://github.com/dnbaker/dashing2-experiments).

## Supplementary Material

Supplement 1

Supplement 2
