# Supplementary material for: Genomic sketching with multiplicities and locality-sensitive hashing using Dashing 2
Source: Genome Res. 2023 Jul;33(7):1218–27. doi: 10.1101/gr.277655.123 (PMC10538361; doi:10.1101/gr.277655.123)
Supplement: Supplement 2 [file Supplemental_Materials.pdf]

Supplementary Material for "Genomic sketching with multiplicities and locality-sensitive hashing using Dashing 2"

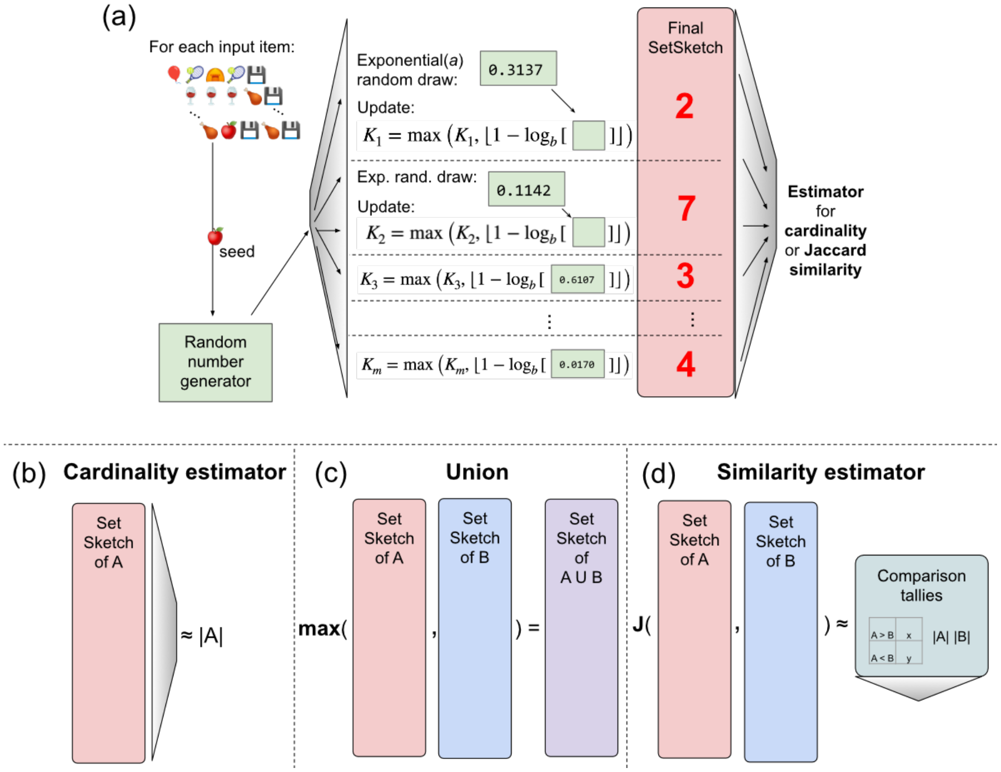

Figure S1: (a) Schematic of SetSketch. A given input item is hashed and its hash value is used to seed the pseudo-random number generator (RNG). The RNG generates exponential random draws for each register. Each register is updated according to the given rule. Once fully populated, the register values are used in combination with different estimators for set cardinality or Jaccard similarity. (b) Illustration of estimating cardinality of set A via its SetSketch. (c) Illustration of combining two SetSketches to obtain the sketch of the uinion. (d) Illustration of using Jaccard similarity estimator to compare two datasets via their SetSketches.

Input: $K[0..m-1]$: SetSketch registers for this dataset
Input: id: Identifier for this dataset
Input: RNG: Pseudo-random number generator
Input: $seed$: Pseudo-random seed
Input: N: Number of super-register sizes in index
Input: LSH: map from ⟨ table id, super-register id, super-register value ⟩ triples to a corresponding list of datasets
Result: LSH updated to include dataset $\langle K, id \rangle$

i ← $N-1$
if $i > 2$ **then**
    RNG.initialize($seed$)
end
// Loop over tables 0 .. N-1, from largest super-register size (most specific)
// to smallest (least specific)
while $i \geq 0$ **do**
    $P \leftarrow \min(2^i, 2i)$
    if $i \leq 2$ **then**
        S ← m/N
    else
        S ← $m \cdot 8/N$
    end
    $j \leftarrow 0$ // Loop over super-registers
    while $j < S$ **do**
        if $i \leq 2$ **then**
            // Next non-overlapping super-register
            SuperReg ← $K[P \cdot j \mathrel{..} P \cdot j + P - 1]$
        else
            // Get uniform random integer in [0, $m - P$]
            ri ← RNG.randomInt(0, $m - P$)
            SuperReg ← $K[ri \mathrel{..} ri + P - 1]$
        end
        // Append this dataset to the list for this table, super-register
        // super-register value combination
        $LSH[\langle i, j$, SuperReg ⟩].append(id)
        $j \leftarrow j + 1$
    end
    $i \leftarrow i - 1$
end

Algorithm S1: Add dataset $\langle K, id \rangle$ to LSH index

Input: SetSketch $K[0..m-1]$, item X
Result: K updated according to X

$\text{RI} \leftarrow \text{hash}(X)$
$q \leftarrow \text{RI} \mod m$
$p \leftarrow \lfloor \text{RI}/m \rfloor$
$K[i] \leftarrow \min(K[i], p)$

Algorithm S2: Update one-permutation Dashing 2 SetSketch. Each K_i gets the minimal 64-bit random draw among draws that map there.

Input : SetSketch $K[0..m-1]$, item X
Output: K' set to a truncated form of K

for $i \leftarrow 0, m-1$ **do**
  $K'[i] \leftarrow \lfloor 1 - \log_b(K[i]) \rfloor$
end

Algorithm S3: Finalize Full Dashing 2 SetSketch

Input : Temporary $K[0..m-1]$ from Algorithm S2
Output: Finalized K'

for $i \leftarrow 0, m-1$ **do**
  $RV \leftarrow -\ln(K[i])$
  $K'[i] \leftarrow \lfloor 1 - \log_b(RV) \rfloor$
end

Algorithm S4: Finalize one-permutation Dashing 2 SetSketch

	Time (seconds)		Memory footprint (MB)	
Method	Sketching	Similarity	Sketching	Similarity
Mash	16.1	4.61	338	41.4
Sourmash	89.4	11.9	141	320
BinDash	26.8	28.2	37.8	124
D2	10.2	0.12	169	39.9
D2-full	16.3	0.13	169	40.2
D2W	47.0	0.13	481	51.7

Table S1: Running time (i.e. wall-clock time) and memory footprint (i.e. peak resident set size) of each sketching tool as measured by `/usr/bin/time -v`. The "Sketching" task involved sketching all 2,020 genome assemblies used in the experiments over 1,010 genome pairs described in Results. The "Similarity" task involved computing all pairwise Jaccard similarities between the sketches produced by the first task. All tools were run with 16 threads. In the case of Sourmash's sketching mode, we used GNU parallel to keep a steady state of 16 simultaneous sketching processes. Smallest and second-smallest values highlighted in red and dark red respectively. Software versions used were Mash v2.3, Sourmash v4.6.1, BinDash v1.0, and Dashing 2 v2.1.6
